# Prevalence, patterns and determinants of dyslipidaemia among South African adults with comorbidities

**DOI:** 10.1038/s41598-021-04150-6

**Published:** 2022-01-10

**Authors:** Charity Masilela, Oladele Vincent Adeniyi, Mongi Benjeddou

**Affiliations:** 1 Department of Biotechnology, University of the Western Cape, Bellville, Cape Town, 7535 South Africa; 2 Department of Family Medicine, Walter Sisulu University, East London, 5200 South Africa

**Keywords:** Diseases, Health care, Medical research

## Abstract

The present study assessed the prevalence, patterns and determinants of dyslipidaemia among South African adults with multi-morbidities. In this study, 614 individuals with DM and hypertension were recruited. Dyslipidaemia was defined as elevated levels of total cholesterol (TC) ≥ 5.2 mmol/L and/or low-density lipoprotein cholesterol (LDL-C) ≥ 2.6 mmol/L, triglycerides (TG) ≥ 1.8 mmol/L and low high-density lipoprotein cholesterol (HDL-C) < 1 mmol/L for men and < 1.2 mmol/L for women. Multivariate regression model (adjusted) analysis was used to identify the significant determinants of dyslipidaemia. The prevalence of dyslipidaemia was 76.7% (n = 471), with females showing the highest prevalence 357 (75.79%). Elevated TG (62.21%) was the most prevalent form of dyslipidemia. Only 103 (16.77%) participants were on statin therapy. The multivariate logistic regression model analysis (adjusted) showed that, the Zulu ethnicity (AOR = 2.45; 95%CI 1.48–4.05) was associated with high TC. DM (AOR = 2.00; 95%CI 1.30–3.06) and the female sex (AOR = 2.54; 95%CI 1.56–4.12) were associated with low HDL-C. Obesity (AOR = 1.57; 95%CI 1.12–2.21) and the Zulu ethnicity (AOR = 1.60; 95%CI 1.00–2.54) were associated with elevated LDL-C. DM (AOR = 2.32; 95%CI 1.61–3.34) was associated with elevated TG. We found a high prevalence of dyslipidaemia. The study further demonstrated that prevention and treatment of dyslipidaemia should be prioritised among individuals with multi-morbidities.

## Introduction

There is an increasing trend in the prevalence of cardiovascular diseases globally as well as in sub-Saharan Africa. This is reportedly a consequence of a surge in the prevalence of cardiovascular risk factors such as hypertension, obesity and diabetes mellitus (DM)^1^. Among the many abnormalities that commonly accompany these diseases are disturbances in the production and clearance of plasma lipoproteins, known as dyslipidaemia^2,3^. Dyslipidaemia has emerged as an important risk factor for cardiovascular diseases, accounting for over 2 million annual deaths and nearly 30 million disabilities world-wide^4^. In the last three decades, the prevalence of dyslipidaemia in sub-Saharan Africa has been stagnant. However, community-based surveys conducted in South Africa reported a prevalence of up to 93.5% in the general public^1,3^. The high burden of dyslipidaemia among South Africans is accompanied by low treatment and control rates of co-morbidities, such as DM and hypertension, as well as an increase in the incidence of cardiovascular diseases^5–7^. Nonetheless, the severity and determinants of dyslipidaemia among South African adults with DM and hypertension is unknown. Therefore, it is important to adequately quantify the prevalence of dyslipidaemia and identify potential influencing factors in order to manage the condition and lower the incidence of cardiovascular diseases.

Dyslipidaemia is characterised by abnormal blood concentrations of one or more of the following: Total cholesterol (TC), low-density lipoprotein cholesterol (LDL-C), high-density cholesterol (HDL-C), and triglycerides (TG)^3^. The onset of lipid profile disturbances is not completely understood; however, studies have shown that dyslipidaemia may develop secondary to metabolic abnormalities such as obesity, hypertension and DM or vice versa. Common features of diabetes-associated dyslipidaemia are low HDL-C, high TG and LDL-C l level, with low HDL-C being the most prominent feature. In obesity, dyslipidaemia is characterised by elevated serum triglyceride, very low-density lipoprotein (VLDL), apolipoprotein B, non-HDL-C levels and normal or slightly elevated LDL-C^8^. The patterns of hypertension-associated dyslipidaemia vary from population to population; however, several studies have identified low HDL-C as the most prominent feature of dyslipidaemia in hypertension^9^), while other studies have reported a high prevalence of mixed dyslipidaemia of high TG and LDL-C^10^.

The importance of treating dyslipidaemia is highlighted in the South African dyslipidaemia guideline consensus statement of 2018. It also states that the cornerstone of dyslipidaemia treatment is lifestyle management, including dietary modifications, physical activity and weight loss (if needed). In addition to lifestyle management, dyslipidaemia can be treated with clinically prescribed drugs including statins^3^. The updated consensus guide to management of dyslipidaemia in South Africa recommends statin therapy to all patients with known cardiovascular diseases risk, including those with DM and familial hypercholesterolemia^3^. Furthermore, statins have been shown to sufficiently lower LDL-C among patients with DM^11^. Among patients with hypertension, statins have been shown to lower plasma levels of LDL-C^12^. While their HDL-C raising ability varies, their overall cardiovascular disease risk lowering properties are well established^13^.

Nevertheless, a negligent health care system that fails to identify high risk patients, fails to initiate statin therapy, is poorly adhered to and lacks educational programs presents a great challenge for the management of dyslipidaemia. Thus, the treatment of dyslipidaemia remains sub-optimal, particularly among individuals with diabetes and hypertension. In addition, the prevalence, patterns and determinants of dyslipidaemia among this sub-group are not well established. The present study bridges these gaps by determining the prevalence, patterns and determinants of dyslipidaemia among South African adults with multi-morbidities.

## Materials and methods

### Ethical approval

Ethical approval for this study was granted by the Ethics Committee of the University of Western Cape (Reference: BM/16/5/19). Permission to implement the study protocol was granted by the clinical governance of the respective hospitals in the Eastern Cape (Cecilia Makiwane Hospital) and Mpumalanga Provinces (Piet Retief Hospital). Participation in this study was voluntary, each participant was issued with an informed consent form as evidence of the voluntary participation. Consenting participants were issued with a research information sheet, made available in three languages (SiSwati, IsiXhosa and IsiZulu), detailing the purpose and process of the study. The rights to privacy and confidentiality of medical information were respected during and after the study, in accordance with the Helsinki Declaration on human and animal research.

### Study design, settings and population

This cross-sectional study was conducted in three primary health care centres (Mkhondo Town Clinic, Thandukukhanya Community Healthcare Center and Piet Retief Hospital) in the rural Mkhondo Municipality of Mpumalanga province and a peri-urban regional hospital in Mdantsane Township (Cecilia Makiwane Hospital) in the Eastern Cape Province, South Africa. The three facilities in Mkhondo serve a combined population of 189,036 people, while Cecilia Makiwane hospital provides both primary and secondary care to the 755,200 residents of Buffalo City Municipality of the Eastern Cape. Briefly, a total of 614 South African adults attending chronic care for diabetes mellitus and hypertension were recruited consecutively between January 2019 to June 2019. A detailed methodology of the study was published elsewhere^14^.

Participants were eligible if they were at least 18 years old, had been on continuous treatment for either DM and/or hypertension for at least a year prior to the study and had been attending regular follow-up visits at any of the four study sites. Pregnant women and individuals who presented with physical impairments that would prevent the measurement of anthropometric indices were excluded from the study. Eligible participants underwent face-to-face interviews conducted by a trained research nurse using a standardised questionnaire which was divided into three major sections, namely, demographic, lifestyle behaviours and clinical data. Furthermore, the nurses measured the weight and height as well as blood pressure (BP) of each participant by using validated tools according to standard protocols. Relevant clinical data such as health status and prescribed drugs were extracted from the medical records of each participant. Physical activity, smoking status, alcohol consumption, fruit and vegetable consumption, and ethnicity were self-reported by each participant.

### Measures

#### Outcome measure

Dyslipidaemia was defined in accordance with the guideline of the Society of Endocrinology, Metabolism and Diabetes of South Africa as elevated levels of TC ≥ 5.2 mmol/L and/or LDL-C ≥ 2.6 mmol/L, TG ≥ 1.8 mmol/L and low HDL-C < 1 mmol/L for men and < 1.2 mmol/L for women^15^.

#### Independent variables

The independent variables in this study were selected based on recent literature^16–18^. The variables were categorised into socio-demographic (residence, ethnicity, physical activity, fast food consumption, fruit and vegetable consumption, oil used in food preparation, alcohol consumption and employment), and clinical (presence of DM, HPT, obesity and current statin use).

Obesity was defined as body mass index (BMI) ≥ 30.0 kg/m^2^ and further categorised as: underweight = BMI < 18.5 kg/m^2^; normal weight = BMI: 18.5–24.9 kg/m^2^ and overweight = BMI: 25.0–29.9 kg/m^2^. Hypertension was defined as blood pressure > 140/90 mmHg and DM status was retrieved from each patient’s medical file.

Employment status was categorised as unemployed and employed. Similarly, alcohol consumption was categorised as never drank or current drinker. Fruit and vegetable consumption was determined using the survey question “do you consume fruits and vegetables? If yes, how often? Fast food consumption was also determined in a similar manner. The responses were categorised as never or 1–3 times/week. The type of oil used during food preparation was categorised as vegetable oil or animal fat (derived from meat products). Physical activity was categorised as active if participants engaged in exercise leading to an increase in heart and respiratory rate, such as gardening, or inactive if they did not engage in any physical activity. Ethnicity was defined as belonging to a social group that has a common language, ancestry or cultural tradition.

### Laboratory assessment

Fasting venous blood samples for lipid assays and glycated haemoglobin were drawn by the research nurse. Blood assays for glycated haemoglobin (HbA1c), TC (TC), low-density lipoprotein (LDL-C), triglyceride (TG) and high-density lipoprotein HDL-C) were conducted by the National Health Laboratory Services (NHLS) of Piet Retief Hospital, Ermelo Regional Hospital and Cecelia Makiwane Hospital in accordance with the standard protocols.

### Statistical analysis

Complete data for 614 participants were captured on Research Electronic Data Capture (Redcap) and analysed by using the IBM SPSS Statistics for Windows, Version 25.0 (IBM Corp., Armonk, New York, USA). The characteristics of the study participants in frequencies and percentages were reported for categorical variables. Associations between the main outcome measure (dyslipidaemia) and participants’ characteristics (socio-demographic and clinical factors) were assessed using multivariate logistic regression (adjusted odds ratios) model analysis with a 95% confidence interval (95% CI) in order to identify the significant and independent determinants of the different markers of dyslipidaemia in the cohort. Age, sex and ethnicity were used as the main adjusting factors. A p-value of less than 0.05 was considered statistically significant.

## Results

### Characteristics of study participants

A total of 614 individuals participated in this study, of whom 75.24% (n = 462) were females and 24.76% (n = 152) were males. About 35.83% (n = 220) of the study participants were aged between 56 and 65 years, 57.17% (n = 351) had a BMI ≥ 30 kg/m^2^ and 43.97% (n = 270) were of Zulu origin. Most study participants were urban residents (75.24%), employed (64.50%), consumed fast food 1–3 times per/week (75.57%) and used vegetable oil in food preparation (85.99%). In addition, 57.65% of the study participants had DM and 95.28% had hypertension (Table 1).

**Table 1:** Socio-demographic and clinical characteristics of the study cohort stratified by sex (n = 614).

Variables	Female (n; %)	Male (n; %)	Overall (n; %)
**All**	462 (75.24)	152 (24.76)	614 (100)
**Age (Years)**			
18–25	01 (0.22)	02 (1.32)	03 (0.49)
26–35	16 (3.46)	04 (2.63)	20 (3.26)
36–45	26 (5.63)	15 (9.87)	41 (6.68)
46–55	96 (20.78)	23 (15.13)	119 (19.38)
56–65	169 (36.58)	51 (33.55)	220 (35.83)
≥ 65	154 (33.33)	57 (37.50)	211 (34.36)
**BMI (kg/m**^**2**^**)**			
< 18.5	07 (1.52)	04 (2.63)	11 (1.79)
18.5–24.9	49 (10.61)	37 (24.34)	86 (14.01)
25.0–29.9	120 (25.97)	46 (30.26)	166 (27.04)
≥ 30	286 (61.90)	65 (42.76)	351 (57.17)
**Ethnicity**			
Swati	66 (14.29)	18 (11.84)	84 (13.68)
Xhosa	171 (37.01)	89 (58.55)	260 (42.35)
Zulu	225 (48.70)	45 (29.61)	270 (43.97)
**Residence**			
Urban	356 (77.06)	130 (85.53)	462 (75.24)
Rural	106 (22.94)	22 (14.47)	152 (24.76)
**Employment**			
Employed	302 (65.37)	94 (61.84)	396 (64.50)
Unemployed	160 (34.63)	58 (38.16)	218 (35.50)
**Alcohol consumption**			
Never Drank	349 (75.54)	52 (34.21)	401 (65.31)
Current Drinker	113 (24.46)	100 (65.79)	213 (34.69)
**Fruit and vegetable consumption**			
1–3 times/ week	449 (97.19)	150 (98.68)	599 (97.56)
Never	13 (2.81)	02 (1.32)	15 (2.44)
**Type of oil used**			
Vegetable oil	394 (85.28)	134 (88.16)	528 (85.99)
Animal fat	68 (14.72)	18 (11.84)	86 (14.01)
**Fast food consumption**			
Never	119 (25.76)	31 (20.39)	150 (24.43)
1–3 times/week	343 (74.24)	121 (79.61)	464 (75.57)
**Physical activity**			
Active	211 (45.67)	100 (65.79)	311 (50.65)
Inactive	251 (54.33)	52 (34.21)	303 (49.35)
**Presence of DM**			
No	253 (54.76)	101 (66.45)	354 (57.65)
Yes	209 (45.24)	51 (33.55)	260 (42.35)
**Presence of hypertension**			
No	21 (4.55)	08 (5.26)	29 (4.72)
Yes	441 (95.45)	144 (94.74)	585 (95.28)

Overall prevalence of dyslipidaemia = 76.7% (n = 471).

### Prevalence and patterns of dyslipidaemia

**Figure 1:**
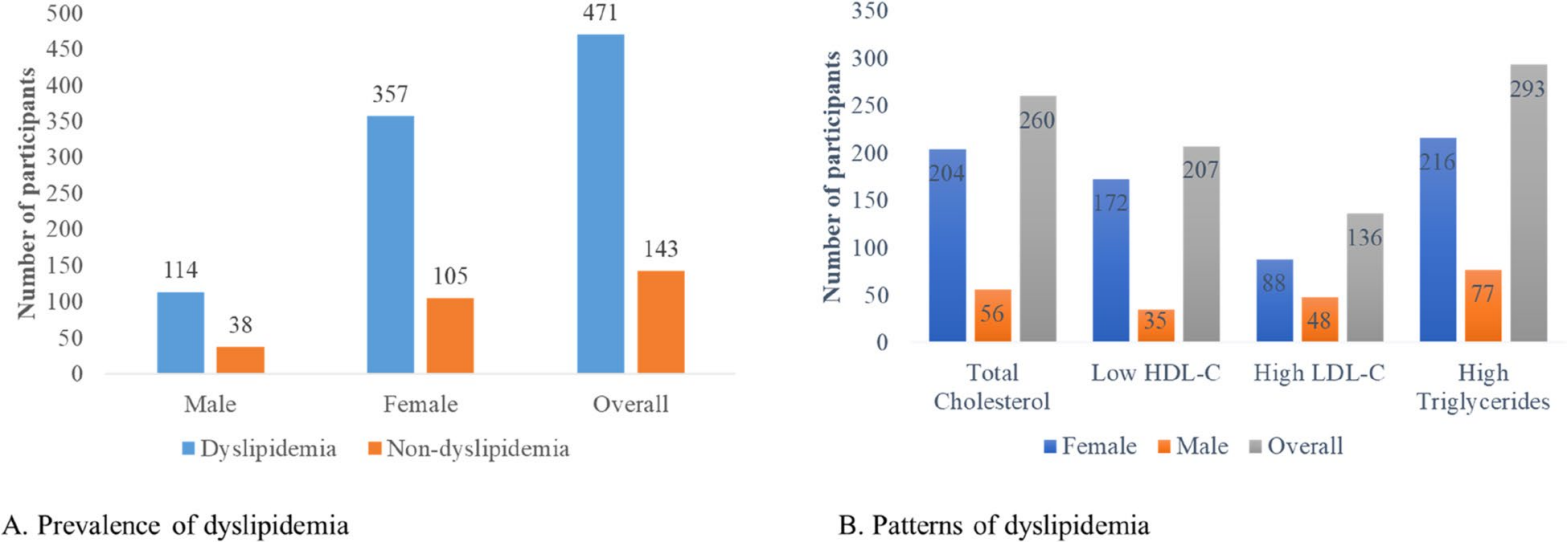
Prevalence and patterns of dyslipidaemia by sex.

The overall prevalence of dyslipidaemia was 76.71% (n = 471) among the study participants. About 75.79% (n = 357) females, 357 met the criteria for dyslipidaemia, while 24.20% (n = 114) males had dyslipidaemia (Fig. 1a). The most prevalent form of dyslipidaemia was elevated TG (62.21%), followed by elevated TC (55.20%), low HDL-C (43.95%) and high LDL-C (28.28%), with women showing the highest prevalence across all categories (Fig. 1b).

**Figure 2:**
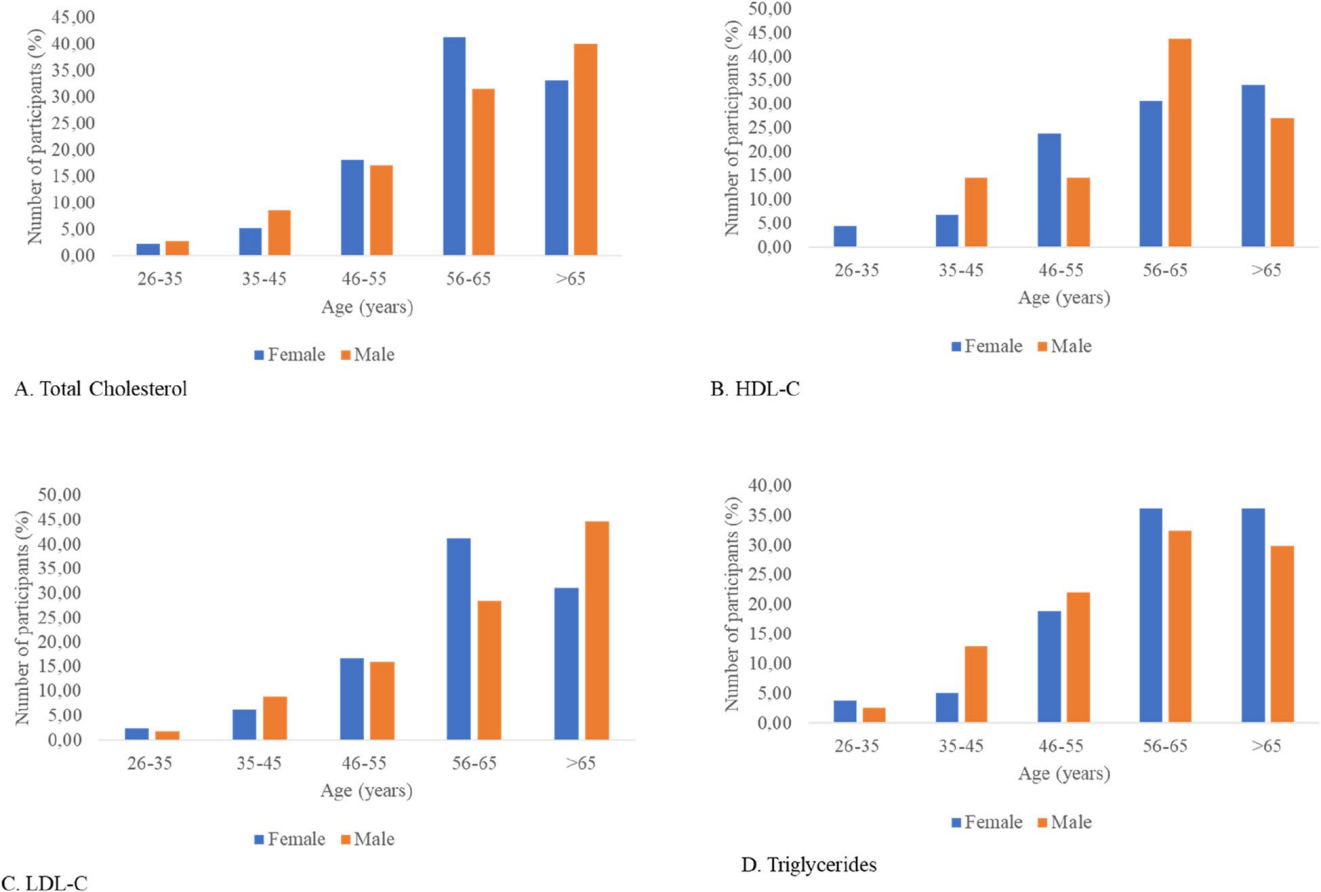
Prevalence of the different types of dyslipidaemias by sex and age group.

The prevalence of elevated TC, TG, LDL-C and low HDL-C increased with age in both men and women, reaching its peak in the age group of 55–65 years (Fig. 2a–d). However, a slight decrease was observed in the age group > 65 years for elevated TC, TG and low HD-LC (Fig. 2a,b,d).

### Patterns of statin prescription

Out of 614 individuals who participated in this study, only 103 (16.78%) were on statin therapy. About 71.84% (n = 74) of the study participants on statin therapy met the criteria for dyslipidaemia, while 28.16% (n = 29) showed normal levels of plasma lipids (results not shown). The rate of statin use among males and females with dyslipidaemia was 4.78% (n = 19) and 11.67% (n = 55), respectively. Furthermore, statin use was higher among participants who were > 65 years of age (n = 47) in comparison to the other age groups (Figure S1). Among individuals who had hypertension, 16.40% (n = 73) were on statin therapy. Whilst 23.08% (n = 51) participants with DM were on statin therapy (Table 2).

**Table 2:** Current statin use among participants with dyslipidaemia (n = 471).

Variable	Current statin use	
	No (n; %)	Yes (n; %)
**Presence of DM**		
No	227 (90.80)	23 (9.20)
Yes	170 (76.92)	51 (23.08)
**Presence of hypertension**		
No	25 (96.15)	01 (3.85)
Yes	372 (83.60)	73 (16.40)

### Factors associated with the different forms of dyslipidaemia

In the multivariate logistic regression model analysis (unadjusted), Participants who were of Zulu [Crude odds ratios (COR) = 1.56; 95% confidence interval (CI) 1.08–2.24] origin as well as individuals who were hypertensive (COR = 1.62; 95%CI 1.09–2.42) were more likely to show elevated TC. While females (COR = 0.52 95%CI 0.34–0.80) and participants who were on statin therapy (COR = 0.49 95%CI 0.30–0.81) were less likely to exhibit elevated TC. Female (COR = 0.49; 95%CI 0.30–0.81) participants were more likely to show low levels of HDL-C, while participants with hypertension (COR = 0.51; 95%CI 0.34–0.77) were less likely to show low HDL-C levels (Table 3).

**Table 3:** Multivariate logistic regression analysis of factors associated with increased TC and decreased HDL-C (n = 614).

Variable	Total Cholesterol			HDL-C		
	Elevate (n; %)	Unadjusted odds Ratios	Adjusted Odds Ratios	Low (n; %)	Unadjusted Odds Ratios	Adjusted Odds Ratios
**Sex**						
Male	36 (23.68)	1	1	48 (31.58)	1	1
Female	172 (37.23)	0.52 (0.34–0.80)**	0.48 (0.30–0.78)**	88 (19.05)	1.95 (1.29–2.95)***	2.54 (1.56–4.12)***
**Age (Years)**						
≤ 45	17 (26.56)	1	1	17 (26.56)	1	1
> 45	191(34.73)	0.79 (0.54–1.16)	1.43 (0.78–2.64)	119 (21.64)	0.77 (0.51–1.16)	0.85 (0.46–1.60)
**Ethnicity**						
Swati	29 (34.52)	1	1	22 (26.19)	1	1
Xhosa	101 (38.85)	0.47(0.72–2.01)	1.35 (0.78–2.31)	55 (21.15)	0.95 (0.63–1.42)	1.45 (0.80–2.65)
Zulu	78 (28.89)	1.56(1.08–2.24)**	2.45 (1.48–4.05)***	59 (21.85)	0.75 (0.42–1.33)	0.78 (0.45–1.37)
**Residence**						
Urban	165 (33.95)	1	1	105 (21.60)	1	1
Rural	43 (33.59)	0.98 (0.65–1.48)	0.96 (0.62–1.48)	31 (24.22)	1.16 (0.73–1.83)	1.07 (0.65–1.75)
**Employment**						
Employed	139 (35.10)	1	1	79 (19.95)	1	1
Unemployed	69 (31.65)	0.85 (0.60–1.21	0.75 (0.52–1.09)	57 (26.15)	1.42 (0.92–2.09)	1.47 (0.97–2.23)
**Alcohol consumption**						
Never Drank	139 (34.66)	1	1	84 (20.95)	1	1
Current Drinker	69 (32.39)	1.10 (0.77–1.57)	1.05 (0.70–1.57)	52 (24.41)	1.21 (0.82–1.80)	0.95 (0.59–1.49)
**Fruit and vegetable consumption**						
1–3 times/ week	202 (33.72)	1	1	130 (21.70)	1	1
Never	06 (40.00)	0.76 (0.26–2.17)	1.44 (0.48–4.31)	06 (40.00)	2.40 (0.84–6.88)	2.33 (0.74–7.26)
**Type of oil used**						
Vegetable oil	184 (34.85)	1	1	120 (22.73)	1	1
Animal fat	24 (27.91)	1.38 (0.83–2.28)	0.73 (0.43–1.24)	16 (18.60)	0.77 (0.43–1.38)	0.83 (0.54–1.52)
**Fast food consumption**						
Never	49 (32.67)	1	1	28 (18.67)	1	1
1–3 times/week	159 (34.27)	0.93 (0.62–1.37)	0.92 (0.59–1.43)	108 (23.28)	1.32 (0.83–0.23)	1.41 (0.84–2.35)
**Physical activity**						
Active	107 (34.41)	1	1	69 (22.77)	1	1
Inactive	101 (33.33)	1.04 (0.75-.46)	1.35 (0.86–2.10)	67 (21.54)	1.07 (0.73–1.57)	1.01 (0.61–1.65)
**Presence of DM**						
No	124(35.03)	1	1	63 (17.80)	1	1
Yes	84 (32.31)	1.18 (0.84–1.67)	0.96 (0.66–1.40)	73 (28.08)	1.38 (0.87–2.18)	2.00 (1.30–3.06)***
**Presence of hypertension**						
No	08 (27.59)	1	1	08 (27.59)	1	1
Yes	200 (34.19)	1.62 (1.09–2.42)**	1.14 (0.46–2.82)	128 (21.88)	0.51 (0.34–0.77)***	1.14 (0.46–2.84)
**Obesity**						
No	79 (30.04)	1	1	51 (19.39)	1	1
Yes	129 (36.75)	1.35 (0.96–1.90)	1.33 (0.93–1.91)	85 (24.22)	1.32 (0.89–1.96)	1.49 (0.98–2.25)
**Current statin use**						
No	184 (36.01)	1	1	110 (21.53)	1	1
Yes	24 (23.30)	0.49 (0.30–0.81)**	0.45 (0.27–0.77)**	26 (25.24)	0.69 (0.43–1.11)	1.01 (0.59–1.73)

**p-values < 0.01; ***p-values < 0.001.

Furthermore, Zulu (COR = 1.50; 95%CI 1.60–2.12), physically inactive (COR = 1.39; 95%CI 1.00–1.91) and individuals with obesity (COR = 1.61; 95%CI 1.61–2.23) were more likely to exhibit elevated levels of LDL-C. Participants who had DM (COR = 2.21; 95%CI 1.55–2.99), physically inactive (COR = 1.40 95%CI 1.02–1.92), as well as those who did not consume fruits and vegetables (COR = 4.49; 95%CI 1.25–16.09) were more likely to show elevated triglyceride levels. On the other hand, individuals who had hypertension (COR = 0.39; 95%CI 0.17–0.88) and belonged to the Zulu (COR = 0.43; 95%CI 0.26–0.72) and Xhosa (COR = 0.52; 95%CI 0.36–0.73) ethnic groups were less likely to show elevated triglyceride levels (Table 4).

**Table 4:** Multivariate logistic regression analysis of factors associated with increased LDL-C and TG levels (n = 614).

Variables	LDL-C			Triglycerides		
	Elevate (n; %)	Unadjusted odds ratios	Adjusted odds ratios	Elevate (n; %)	Unadjusted odds ratios	Adjusted odds ratios
**Sex**						
Male	56 (36.84)	1	1	77 (50.66)	1	1
Female	204 (44.16)	0.74 (0.50–1.08)	0.78 (0.50–1.19)	217 (46.97)	1.16 (0.80–1.68)	1.47 (0.96–2.26)
**Age (Years)**						
≤ 45	67 (36.81)	1	1	91 (50.00)	1	1
> 45	193 (44.68)	1.38 (0.97–1.97)	1.18 (0.68–2.06)	203 (46.99)	0.88 (0.62–1.25)	1.07 (0.61–1.88)
**Ethnicity**						
Swati	32 (38.10)	1	1	49 (58.33)	1	1
Xhosa	125 (48.08)	1.50 (0.91–2.48)	1.04 (0.62–1.75)	99 (38.08)	0.52 (0.36–0.73)***	0.49 (0.30–0.78)**
Zulu	103 (38.15)	1.50 (1.60–2.12)**	1.60 (1.00–2.54)*	146 (54.07)	0.43 (0.26–0.72)***	1.34 (0.79–1.26)
**Residence**						
Urban	207 (42.59)	1	1	233 (47.94)	1	1
Rural	53 (41.41)	0.95 (0.64–1.14)	0.98 (0.65–1.48)	61 (47.66)	0.98 (0.66–1.46)	0.80 (0.53–1.22)
**Employment**						
Employed	170 (42.93)	1	1	191 (48.23)	1	1
Unemployed	90 (41.28)	0.93 (0.66–1.30)	0.84 (0.59–1.20)	103 (47.25)	0.96 (0.69–1.33)	1.06 (0.74–1.51)
**Alcohol consumption**						
Never Drank	174 (43.39)	1	1	188 (46.88)	1	1
Current Drinker	86 (40.38)	0.88 (0.63–1.23)	0.89 (0.61–1.30)	106 (49.77)	1.12 (0.85–1.40)	1.19 (0.81–1.71)
**Fruit and vegetable consumption**						
1–3 times/ week	253 (42.24)	1	1	282 (47.08)	1	1
Never	07 (46.67)	1.19 (0.42–3.34)	1.56 (0.53–4.53)	12 (80.00)	4.49 (1.25–16.09)*	3.33 (0.88–12.51)
**Type of oil used**						
Vegetable oil	225 (42.61)	1	1	252 (47.73)	1	1
Animal fat	35 (40.70)	0.94 (0.58–1.46)	0.95 (0.59–1.55)	42 (48.84)	1.04 (0.66–1.64)	0.96 (0.59–1.55)
**Fast food consumption**						
Never	58 (38.67)	1	1	81 (54.00)	1	1
1–3 times/week	202 (43.53)	1.22 (0.83–1.78)	1.07 (0.71–1.62)	213 (45.91)	0.72 (0.50–1.04)	0.89 (0.58–1.34)
**Physical activity**						
Active	116 (38.28)	1	1	158 (52.15)	1	1
Inactive	144 (46.30)	1.39 (1.00–1.91)*	0.85 (0.56–1.29)	136 (43.73)	1.40 (1.02–1.92)*	0.89 (0.59–1.36)
**Presence of DM**						
No	146 (41.24)	1	1	141 (39.83)	1	1
Yes	114 (43.85)	0.86 (0.57–1.30)	1.27 (0.89–1.82)	153 (58.85)	2.21 (1.55–2.99)***	2.32 (1.61–3.34)***
**Presence of hypertension**						
No	08 (27.59)	1	1	20 (68.97)	1	1
Yes	252 (43.08)	1.12 (0.78–1.16)	1.97 (0.81–4.79)	274 (46.84)	0.39 (0.17–0.88)*	0.67 (0.28–1.61)
**Obesity**						
No	94 (35.74)	1	1	120 (45.63)	1	1
Yes	166 (47.29)	1.61 (1.61–2.23)**	1.57 (1.12–2.21)**	174 (49.57)	1.17 (0.85–1.16)	1.30 (0.92–1.84)
**Current statin use**						
No	222 (43.44)	1	1	248 (48.53)	1	1
Yes	38 (36.89)	1.08 (0.70–1.65)	0.60 (0.37–0.96)*	46 (44.66)	1.08 (0.71–1.65)	0.76 (0.47–1.21)

*p-values <0.05; **p-values <0.01; ***p-values<0.001.

In the multivariate logistic regression model analysis (adjusted), females [adjusted odds ratio (AOR) = 0.48; 95% confidence interval (CI) 0.30–0.78] and individuals on statin therapy (AOR = 0.45; 95%CI 0.27–0.77) were less likely to exhibit elevated TC, while Zulu participants (AOR = 2.45; 95%CI 1.48–4.05**)** were more likely to exhibit elevated TC. Individuals with DM (AOR = 2.00; 95%CI 1.30–3.06) and females (AOR = 2.54; 95%CI 1.56–4.12) were more likely to have low HDL-C (Table 3).

Participants who were obese (AOR = 1.57; 95%CI 1.12–2.21) as well as those who belonged to the Zulu tribe (AOR = 1.60; 95%CI 1.00–2.54) were more likely to have elevated levels of LDL-C, whereas participants who were on statin therapy (AOR = 0.60; 95%CI 0.37–0.96) were less likely to show elevated LDL-C levels. In addition, participants with DM (AOR = 2.32; 95%CI 1.61–3.34) were more likely to have elevated TG. Participants belonging to the Xhosa tribe (AOR = 0.49; 95%CI 0.30–0.78) were less likely to show high levels of TG (Table 4).

## Discussion

Dyslipidaemia is a serious medical condition that causes and worsens co-morbidities, increases the incidence of cardiovascular diseases and mortality. However, the burden of dyslipidaemia and potential risk factors among South African adults with DM and hypertension are not well established. Therefore, this study bridges the gap by reporting on the prevalence, patterns and the associated risk factors of dyslipidaemia among South African adults with multi-morbidities. Atherogenic dyslipidaemia, hyperglycemia, visceral obesity and hypertension tend to occur in clusters in the same individuals. As such, the high prevalence of dyslipidaemia (76.7%) reported in this study is consistent with this expectation. Our result is in line with observations made among patients with DM and hypertension across African countries such as Uganda (88%)^19^, Ethiopia (71.2%)^17^, Kenya (86.1%)^20^ and Nigeria (72.4%)^21^. In comparison to observations made across South Africa among patients with DM and/or hypertension, the prevalence reported in the current study was slightly lower than the 89% observed in Cape Town^22^, 90% observed in Durban^23^ and 93.5% that was reported in Johannesburg^24^. In addition, countries such as China (31.8%)^18^ and Germany (64.5%)^25^ reported a lower prevalence of dyslipidaemia in individuals with DM and/or hypertension in comparison to the present study.

The most prominent form of dyslipidaemia was elevated TG (62.21%), followed by elevated TC (55.20%), with women showing the highest prevalence of all measures of dyslipidaemia in comparison to men. The patterns observed in the prevalence of dyslipidaemia differ from observations reported by Asiki et al.^26^, Ayoade et al.^14^, and Gebreegziabiher et al.^27^, where the most prevalent dyslipidaemia markers were HDL-C, TC and LDL-C, respectively. Although, there were disparities observed in the prevalence of the different measures of dyslipidaemia, most reference studies reported a high prevalence of all forms of dyslipidaemia among women.

In addition, the present study showed that the prevalence of all forms of dyslipidaemia increased with age in both men and women but was slightly decreased among older adults (> 65 years). This may be a result of the high rate of statin prescription that was observed among the age group. Similar observations were reported among Chinese patients^18^ with DM and hypertension. Although, dyslipidaemia is reportedly common among patients with both DM and hypertension, the disease was thought to be rare among Black Africans, possibly due to genetic, nutritional and environmental factors. However, the current study contradicts previous perceptions, and it has clearly shown that dyslipidaemia is highly prevalent among Africans. This study has also shown that the patterns of dyslipidaemia prevalence vary according to ethnic group, health status, sex and age group in the country.

Furthermore, the present study showed that women were less likely to exhibit high levels of TC. Similarly, a study conducted among Chinese adults with hypertension showed that women were more likely to have low levels of cholesterol in comparison to their male counterparts^28^. However, a study conducted in Saudi Arabia further demonstrated that females were 1.4 times more likely to have high levels of TC^29^. Similar observations were also reported by Shohaimi et al.^30^ and the China National Stroke Screening and prevention project (CNSSPP)^31^. It is important to note that the reference studies were composed of mixed cohorts of healthy individuals and others with comorbidities (DM /or hypertension), while the present study was composed of individuals with co-morbidities (DM and/or hypertension). Differences in sampling methods may be the reason for the disparities observed.

This study further demonstrated that women were more likely to have low levels of HDL-C. In contrast, a study conducted by Ge et al.^32^ showed that men were more likely to have low HDL-cholesterol. Similar observations were also reported by Lazo-Porras et al.^33^ in Peru, Kim et al.^1^ in South Korea and Ge et al.^32^ in China. On the other hand, Agongo et al.^11^ showed that there was no association between sex and low HDL-C among Ghanaian patients with both DM and hypertension. Ayoade et al.^16^ showed that there was a significant relationship between gender and lipoproteins among Nigerian participants with hypertension, with women showing a higher prevalence of low HD-LC. It is widely accepted that HDL-C concentrations vary with sex, and women naturally have high levels of HDL-C. However, it appears that drastic changes occur in the cases of diabetes or hypertension.

Epidemiological studies have indicated that that there are major differences in cardio-metabolic risk factors between ethnic groups, and that the differences may be reflected in the patterns of dyslipidaemia. These disparities may be attributed to a variety of factors including lifestyle, genetic and cultural factors. In the current study, participants of Zulu origin were more likely to have high levels of TC and LDL-C. On the other hand, participants of Xhosa origin were less likely to exhibit high levels of TG. Ethnical differences in the patterns of dyslipidaemia were also reported by Frank et al.^34^ among individuals of Asian origin, where Asian Indian, Filipino and Vietnamese participants had a higher risk of possessing all three dyslipidaemia subtypes (Low HDL-C, High LDL and TG), whereas Japanese participants were more likely to show combined dyslipidaemia characterised by high TG and low HDL-C. In addition, a study conducted in Uganda among individuals belonging to the Rwandese, Muganda and other tribes, showed that the Rwandese tribe had higher odds of low HDL-C, while individuals in the non-Rwandese tribe had higher odds of elevated TC^26^. Unfortunately, there is no record detailing the patterns of dyslipidaemia across the different ethnic groups in South Africa and their association with each of the tribes. Future studies should focus on exploring the ethnically diverse population of South Africa.

In a healthy state, HDL-C exerts various antiatherogenic properties by reversing cholesterol transport, anti-oxidative and anti-inflammatory capacities, while TG serve as an important part of energy storage. According to the literature, DM particularly type 2 (T2DM), is associated with dyslipidaemia which involves abnormalities in all types of lipoproteins. In this study, the presence of DM was significantly associated with low levels of HDL-C and elevated TG. Similar effects were demonstrated in several studies, where concentrations of HDL-C were diminished in patients with DM. Other studies have also demonstrated that patients with DM were more likely to exhibit elevated TG. Changes in triglyceride and HDL-C concentrations among patients with DM may be attributed to insulin resistance, hyperglycaemia and hyperinsulinemia. Insulin resistance is believed to increase the availability of free fatty acids. On the other hand, hyperinsulinemia and hyperglycaemia promote triglyceride synthesis by activating carbohydrate-responsive element-binding protein sterol regulatory element-binding transcription factor 1 (SREBF1c). These events lead to the activation of cholesterol ester transfer protein (CETP), which is responsible for enhanced HDL-C catabolism^35^. We did not report on the proportion of glycaemic control and the degree of insulin resistance among our study participants; however, it appears that the high prevalence of low HDL-C may be attributed to uncontrolled DM or a high degree of underlying insulin resistance.

Dyslipidaemia is a common feature of the metabolic syndrome including diabetes and obesity. Among obese individuals, the hallmark of dyslipidaemia is elevated TG and FFA, decreased HDL-C with HDL dysfunction and normal or slightly elevated LDL-C. In the present study, obese participants were more likely to show elevated LDL-C. Similarly, a national health survey conducted in Brazil showed that obesity was significantly associated with high levels of LDL-C^36^. It has been suggested that the changes observed in obesity related dyslipidaemia are a result of hypertriglyceridemia, which often leads to a delay in the clearance of triglyceride rich lipoproteins and the formation of small dense LDL-C^37^. Another cause could be enhanced production of apoprotein B-containing lipoproteins among obese individuals, which enhances the conversion of very-low-density lipoprotein (VLDL) to LDL. In this study, we did not quantify the sub-classes of LDL-C; however, the results obtained indicate that treatment of dyslipidaemia should be focused on lifestyle changes including weight loss, physical exercise and a healthy diet. Lifestyle changes will synergistically improve glycaemia and blood pressure.

According to the American Diabetes Association (ADA), statin therapy should be initiated in individuals with DM and other cardiovascular risk factors such as hypertension^38^. Similarly, South Africa’s guidelines for diabetes management state that statin therapy should be added to lifestyle therapy, regardless of baseline lipid levels, for all patients who are either older than 40 years of age or have had diabetes for longer than 10 years, with one or more additional cardiovascular risk factor (hypertension, cigarette smoker, low HDL-cholesterol level, family history of early coronary heart disease, and any albuminuria)^39^. There are no clear guidelines for the treatment of dyslipidaemia among individuals with hypertension in South Africa. However, several studies have shown that hypertension contributes to overall cardiovascular risk, and that the use of a statin should be considered in hypertensive patients with low-moderate cardiovascular diseases risk^40^. In this study, most of the participants were hypertensive; however, only 16.40% of this sub-group were initiated on statin therapy. Among those with DM, only 23.08% were on statin therapy. Despite the low number of patients on lipid-lowering medication, participants who were on statin therapy were less likely to have elevated TG and TC.

Although the SEMDSA guidelines recommend prescribing lipid-lowering medication to all patients with type 2 diabetes mellitus, including those with co-existing DM and hypertension, this data indicates that compliance with these recommendations remains poor. This has led to poor risk factor management and an increased risk of adverse outcomes. Plausible explanations for the observed poor compliance with clinical guidelines could be due to doctors’ inertia to initiate and optimise statin therapy^41^. Several factors such as non-adherence and adverse effects associated with long term statin use could justify failure to initiate this treatment in individuals with comorbidities.

In addition, this study also investigated lifestyle factors that are related to the development of dyslipidaemia (physical activity, fruit and vegetable intake, fast food consumption, type of oil used in food preparation and alcohol consumption). However, none of the investigated factors were associated with dyslipidaemia in this study. These observations differ from previous reports which showed that alcohol consumption was associated with elevated triglyceride, LDL-C and low HDL-C^42,43^. Other studies showed that frequent consumption of fast food (French fries) and snacking in between meals significantly increased TC, TG and LDL-C^42^ whilst a diet composed of whole grains, vegetables and fruits was associated with a protective effect against hypertriglyceridemia^44,45^. A recent systematic review showed that coconut oil consumption results in significantly higher LDL-C than non-tropical vegetable oils^46^. In contrast, Khaw et al.^47^ showed that animal derived fat (butter) significantly increased LDL-C, whereas coconut oil improved HDL-C level. Literature supports the beneficial effects of plant-derived oils on dyslipidaemia; however, it appears that their positive effects may depend on the kind of food that is being prepared.

While several studies have reported that physical activity had no effect on the incidence of dyslipidaemia, others have shown that individuals who engaged in physical activity were less likely to exhibit high levels of TG and low HDL-C^48,49^. It is important to note that lifestyle measures were obtained by self-report. While self-report is a convenient method for data collection, the results are subjective. More studies assessing the association between lifestyle factors and dyslipidaemia are needed in order to support these findings and therefore allow for generalisation of the observations.

### Study limitations

This study highlights the high prevalence of dyslipidaemia and its associated risk factors among ethnically diverse South African adults from the Eastern Cape and Mpumalanga provinces. However, the limitations of the study cannot be ignored: the lack of information on measures such as waist circumference, waist-to-hip ratio and family history; self-report of important lifestyle variables; cross-sectional design and the convenience sampling method which could be subjective. As such, the findings are generalisable only to the population of individuals living within the two regions and similar settings in the country.

## Conclusion

In the current study, we found a high prevalence of dyslipidaemia, with the most common form being elevated TG. We also observed a high prevalence of the different markers of dyslipidaemia among women. In addition to the high prevalence among women, we observed that the prevalence of the different forms of dyslipidaemia increases with age in both men and women. Moreover, factors associated with dyslipidaemia as defined by elevated TC, and/ or high LD-C, high TG and low HD-LC were sex (female), presence of DM, obesity, ethnicity (Zulu) and current statin use. Thus, the incidence of dyslipidaemia could be reduced, and its progression slowed by controlling these factors. Furthermore, lipid-lowering medication was underutilised among the study population, possibly as a result of doctors’ inertia to initiate and optimize statin therapy, or patients’ reluctance to accept lipid-lowering medication. These findings have indicated that health education should be promoted among patients in order to improve dyslipidaemia awareness and promote the use of lipid lowering medication among high-risk patients. Overall, the findings of this study have shown that the prevention and treatment of dyslipidaemia should be prioritised in the at-risk population of the country.

## Supplementary Information

Supplementary Information.

## Data Availability

The data presented in this study is available from the corresponding author upon reasonable request.
