# Supplementary figures and images for: Prevalence, patterns and determinants of dyslipidaemia among South African adults with comorbidities
Source: Sci Rep. 2022 Jan 10;12:337. doi: 10.1038/s41598-021-04150-6 (PMC8748924; doi:10.1038/s41598-021-04150-6)

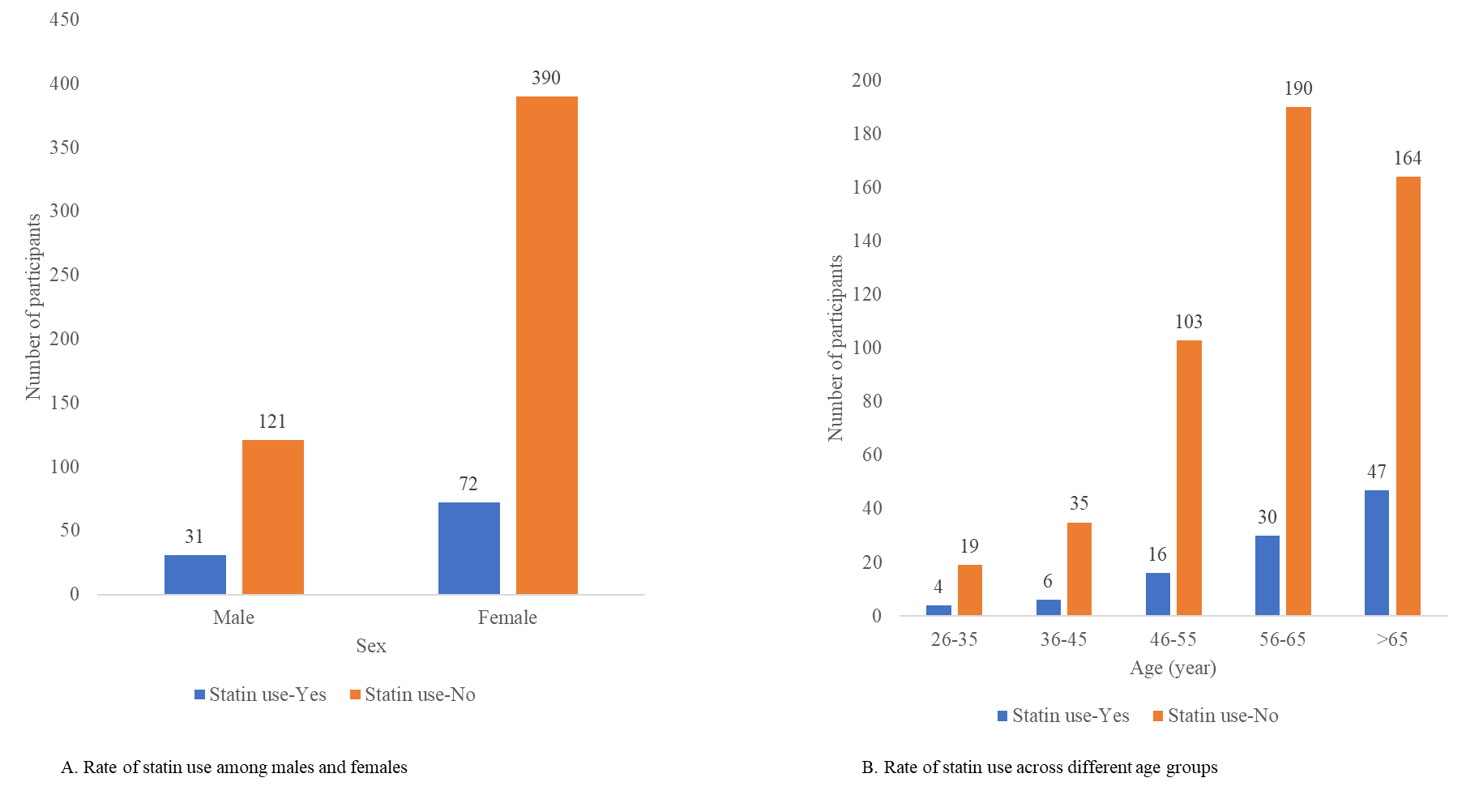


**S1: Current statin use by Sex and age**

Supplement: Supplementary file 1 — Supplementary Information. [file 41598_2021_4150_MOESM1_ESM.docx]
